# Modeling genetic components of hatch of fertile in broiler breeders

**DOI:** 10.1016/j.psj.2021.101062

**Published:** 2021-02-19

**Authors:** Bayode O. Makanjuola, Victor E. Olori, Raphael A. Mrode

**Affiliations:** ∗ Centre For Genetic Improvement of Livestock, Department of Animal Biosciences, University of Guelph, Guelph, Ontario N1G 2W1, Canada; † Aviagen Limited, Newbridge, EH28 8SZ Scotland, United Kingdom; ‡ Livestock Genetics Program, International Livestock Research Institute, Nairobi, Kenya; § Animal and Veterinary Science, Scotland Rural College, Roslin Institute Building, Easter Bush EH15 9RG, Scotland, United Kingdom

**Keywords:** broiler breeder, hatch of fertile, random regression, modeling

## Abstract

Reproductive efficiency such as fertility and hatch of fertile (**HoF**) are of economic importance and concern to breeding companies becaue of their effects on chick output. Similar to other traits of economic importance in poultry breeding, the rate of response for HoF is largely dependent on the use of an appropriate model for evaluating the trait. Therefore, the objectives of this study were to estimate genetic parameters from cumulative, repeatability, fixed regression, random regression, and multitrait models for HoF from a pure-line broiler breeder. The data available for this study consisted of weekly HoF records from 11,729 hens with a total pedigree record of 38,260. Estimates of heritability from the various models ranged from 0.04 to 0.22 with the highest estimate obtained from the cumulative model and the lowest from the repeatability model. Responses to selection estimated for the different models ranged from 0.03 to 0.08% gain per year of the phenotypic mean. In general, the cumulative and the repeatability models underestimated response to selection. The multitrait and random regression models gave similar results for response to selection at 0.08 percentage change in phenotypic mean. In conclusion, the cumulative model is not optimal for modeling HoF, and likewise, the repeatability model. The random regression and multitrait models should be considered instead as they offered a higher response to selection. However, if a multitrait analysis is to be considered, it is recommended to split up the production period in such a way as to avoid computational constraints due to overparameterization.

## Introduction

Poultry production has undergone intensive development with various biosecurity techniques, improved health facilities, increased production, and advanced genetics methodologies put in place since the 1940s (Permin and Pedersen, 2000). With these developments and ongoing research, poultry has been reported to account for more than 30% of animal protein (FAO, 2006) and is expected to account for 41% of expected total growth in meat production by 2027 (OECD/FAO, 2018), which is far more than any other livestock species (Executive Guide to World Poultry Trends, 2019). The growth witnessed in poultry could be attributable to the reduced time taken for broiler chickens to reach the market weight of 1.5 kg, which was 120 d in 1925 and 33 d in 1998 (Decuypere et al., 2003).

The persistence of this improvement is largely due to chick output, which can be attributed to reproductive efficiency. However, improvement in growth rate could have an adverse effect on functional traits such as fertility and hatchability (Chambers, 1990) because of the existence of antagonistic genetic relationship. With the knowledge of this negative correlations, breeding companies have used the multivariate models that coevaluate growth and functional traits to allow improvement of production traits without any trade-off in the welfare of the animals (Laughlin, 2007; Kapell et al., 2012a; Avendaño et al., 2017). More recently, emphases are placed on modeling fitness traits by using a single trait or multitrait model (**MTM**) and fitting known fixed and random factors that have an effect on the traits to properly structure and improve the traits independently. The fitting of optimal models to estimate genetic parameters and design breeding programmes is crucial to improve the accuracy of selection. Some models such as animal repeatability (König et al., 2006), fixed and random regression models (**RRM**) (Ptak and Schaeffer, 1993; Schaeffer, 2004; Wolc et al., 2009, 2010) have been used in the estimation of genetic parameters of various traits in different livestock species.

The production of chicks is dependent on a chain of traits such as egg production, which is predominantly regarded as a trait of the dam; egg fertility, which is influenced mostly by the male (sire) that mated with the hen and the hen herself (dam); and egg hatchability. In a broad sense, hatchability may be defined with reference to all the eggs set in the incubator (hatch of set [HoS]), which is influenced by the genetic and nongenetic effects of both the hen and her mate through fertility. On the other hand, in a narrow sense, hatch of fertile (**HoF**) is defined with reference to only the number of fertile eggs, and this is mostly a trait of the hen (egg quality) and the genotype of the embryo (Wolc et al., 2009).

Egg production is an important economic trait in both layers (egg-type) and broiler (meat-type) breeders; however, genetic parameters estimation for this trait using different models has focused more on the egg-type breeder (Anang et al., 2002; Wolc et al., 2007a, Wolc et al., 2007b) with only a few on the meat-type breeder (Koerhuis and McKay, 1996; Luo et al., 2007). Traditionally, this trait has been modeled as a single trait in terms of the cumulative egg number of the hen over the productive cycle for up to 40 wk. Thus, each individual animal has only one record for their production period. This model is similar to the 305-day model used in dairy cattle milk production and does not consider the effect of changes in the permanent environment due to the longitudinal nature of the trait. Swalve (1995) estimated higher heritability for the cumulative 305-day model than that for the test day model which includes a permanent environmental term. Although not directly comparable, the lactation curve in dairy cattle and egg production curve in poultry follow a similar trend with an increase in the first period up to a peak after which there is decline in the production (Wolc et al., 2007a). This trend indicates that different time points in egg production of the animals could be used to evaluate the persistency of egg production. With the widespread usage of test day model in dairy cattle production, which more accurately captures the pattern of the lactation curve, there have been awareness to implement weekly or monthly cumulative record models for egg production trait to better capture the trend in the production curve using fixed (Anang et al., 2001a) and RRM (Anang et al., 2002; Luo et al., 2007; Wolc and Szwaczkowski, 2009).

Hatchability in broiler breeders is a trait that determines chick output; therefore, it is an economically important trait in poultry meat production. Hatchability is a reproductive trait that is affected by different factors most of which have been reported in literatures. These include the age of the hen (Lapão et al., 1999), egg size (Abiola et al., 2008), nutrition of the dam (Wilson, 1997), and the storage length of the laid eggs (Heier and Jarp, 2001). In a review by Wolc et al. (2010), a positive correlation was found between fertility and HoS indicating that HoS is a trait influenced by sire and dam components, with the sire component more significant than that of the dam because infertile eggs were not broken up to determine whether it resulted from true infertility or early embryo death. In other words, early embryo death and true infertility were classified as sire effects. Conversely, HoF was predominantly determined as the trait of the dam, which is due to the environment provided for the development of the embryo. With these findings, it is evident that HoS is correlated to both fertility and HoF. Owing to the importance of chick output and the need to explore fertility and hatchability independently, this study primarily focuses on HoF rather than HoS.

The acceptance of the cumulative record model in poultry breeding to date can be attributed to the rapid increase in genetic improvement achieved with selection based on the animal's early records and the short generation interval (VanVleck and Doolittle, 1964). However, this model does not account for the longitudinal nature of the trait and thus causing heritability to be upwardly biased (Wolc et al., 2007b). This inadequacy has led to the use of monthly records as a repeated measurement of the same trait or as separate traits (Anang et al., 2001b). Repeatability model (**REP**) assumes a correlation of unity between all the records and a constant heritability throughout the production period. Nonetheless, this does not account for the possibility that different genes may be expressed at different times in the production cycle of an animal (Liljedahl et al., 1999). A MTM that classifies repeated measurements of the same trait on an animal as different traits has been implemented in the estimation of genetic parameters. However, this model is cumbersome because it could involve the use of a large amount of data, a large number of traits, and overparameterization of the data to analyze (Anang et al., 2001b). The RRM has been widely implemented for longitudinally recorded traits with evidential success in dairy, beef, goat, and poultry production (Santana et al., 2015; Brito et al., 2017; Kheirabadi, 2018; Miyumo et al., 2018; Padilha et al., 2019). This model makes use of repeated measurement of the same trait but accounts for the longitudinal nature of the trait. It usually involves fitting a fixed productive curve for all animals or groups of animals reared together. This is achieved by modeling the production curve parameters on a variable such as age. A fixed production curve fitted in the usual REP is termed fixed regression model (**FRM**) (Ptak and Schaeffer, 1993). However, modeling each individual by means of a random regression curve in addition to the fixed productive curve is termed as the RRM (Schaeffer and Dekkers, 1994). The RRM is more preferred because of the ability to more precisely adjust for the environmental effects occurring over the duration of the production period. In addition, RRM may require the estimation of fewer parameters in comparison to the MTM.

The objectives of this study sought to 1) compare the genetic parameters estimated from cumulative, repeatability, multitrait, and RRM for HoF in broiler breeders and 2) estimate the response to selection from each of the models.

## Materials and methods

### Data Collection

Data on weekly HoF and pedigree information from 38,260 hens of a broiler breeder pure line were provided by Aviagen Ltd. (a poultry breeding company in the United Kingdom). Eggs were collected twice daily and marked to indicate the hen that laid the eggs and her mates. Collected eggs were stored in an egg room with optimum temperature and humidity set according to standard practice to prolong the shelf life of fertile eggs before being transferred to the incubator (setter). This implied that depending on the day the eggs were laid and set in the incubator, some hens had their eggs transferred fresh while others were stored for various number of days up to a maximum of 13 d. Storage for 10 to 13 d was more frequent in the later ages of the hen productive life when egg production drops with hens having longer days without laying eggs. Eggs that were cracked, oversized, and irregularly shaped were not set in the incubator because they were prone to spoilage or finding it difficult to fit into the incubator tray. Incubated eggs were candled on the seventh day to check for fertility. Candling is a process of passing light through the eggs to check the internal features; clear eggs are known to be infertile while fertile eggs are indicated by the presence of a small reddish area (blood), which is the embryo. Fertility as a trait is measured as the proportion of total number of eggs set that were fertile, and HoF is the proportion of fertile eggs that hatched into chicks. Around the 17th day, incubated eggs are moved from the setter to the hatcher in preparation for hatching into chicks. The percent HoF was estimated as follows:

$$\text{\%HoF}=\frac{\text{Number of hatched eggs}}{\text{Number of fertile eggs }}\;\times \;100$$

### Data Editing

Data editing was carried out in R statistical programme (R Core Team, 2012). The raw data consisted of 30,739 hens with a total record of 481,397. Weekly records with no egg produced for setting were removed from the data because this resulted in no chick output and caused missing values for HoF. The removal of these records will have no substantial influence on the genetic parameter estimates of HoF; however, this will considerably affect egg production as a trait, which was not considered in this study. In addition, unmarked eggs with no properly identified hen may result in weeks with no egg produced for setting, and including these records could downwardly bias the estimates of those animals. In order to reduce computational time and to allow for a faster convergence of the different models used in this study, the data were truncated to span a period of 7 yr (2006–2012). In doing this, all birds had their first record starting from 2006, and any bird with records that overlapped from the previous year were removed. In addition, hens with less than 5 records were removed to account for birds that died early in the production cycle. The new data used for further analyses consisted of 188,099 weekly records from 11,729 hens ranging in age from 27 to 58 wk when the eggs were hatched.

### Data Structure

The data consisted of a variable called POU, which is the fixed class describing the contemporary group of birds from 3 successive weeks of hatches that were reared together under the same conditions. In addition, the data contained information on the hen that laid the eggs, the male that mated with the hen, the age of the hen in weeks when her eggs were hatched, and the average age of eggs in days for the number of eggs set per hen per week, percentage of fertilized eggs hatched (HoF). Summary statistics of data is given in Table 1.

**Table 1:** Summary statistics of the data.

Variables	Mean	Standard deviation
Age (week ranging from 28–56)	39.34	6.84
Egg age (day)	3.23	1.36
%HoF	84.40	23.23
Egg set (per hen)	5.63	1.89

Abbreviation: HoF, percentage hatch of fertile.

### Statistical Analysis

All models were analyzed using the ASReml program version 3 (Gilmour et al., 2009).

### Repeatability Model

To investigate the variables that contributed significantly to the variation observed in HoF, an initial simple REP, which included all available fixed and random variables affecting HoF, was fitted. This model assumed a correlation of unity for successive records of the hen during the production period. Subsequently, this allowed for the removal of variables with no significant contribution to HoF, thus reducing overparameterization of the models. The REP fitted was

[1] $$y_{ijkno}=\mu +POU_{i}+Ag_{j}+Eg_{jk}+a_{Mn}+a_{Fno}+pe_{Mn}+pe_{Fno}+e_{ijkno}$$

where $y_{ijkno}$ is the observed record for HoF of hen *o* mated to male *n* at age *j* belonging to class *i* with an average egg age *k*, μ is the mean, *POU*_*i*_ is the *i*^*th*^ fixed class to which the animals belong (both hen and mate), *Ag*_*j*_ is the *j*^*th*^ age of the hen when the records were collected, $Eg_{jk}$ is the *k*^*th*^ average age in days of all eggs set at age *j*, $a_{Mn}$ is the random additive genetic effect of the *n*^*th*^ male, $a_{Fno}$ is the random additive genetic effect of the *o*^*th*^ hen mated to the *n*^*th*^ male, $pe_{Mn}$ is the random permanent environment effect of the *n*^*th*^ male, $pe_{Fno}$ is the random permanent environment effect of the *o*^*th*^ hen mated to the *n*^*th*^ male, and $e_{ijkno}$ is the residual error term. The age of hen and average age of eggs were fitted as covariates. The random additive genetic effect of the male had a nonsignificant contribution on the trait; hence, it was removed from subsequent analyses. The assumptions of the random effects were $a_{Fno}∼N\left(0,\;A\sigma_{a_{Fno}}^{2}\right)$, $pe_{Mn}∼N\left(0,\;I\sigma_{pe_{Mn}}^{2}\right)$, $pe_{Fno}∼N\left(0,\;I\sigma_{pe_{Fno}}^{2}\right)$, $e_{ijkno}∼N\left(0,\;I\sigma_{e_{ijkno}}^{2}\right)$, where $\sigma_{a_{Fno}}^{2}$ is the hen's additive genetic variance, $\sigma_{pe_{Mn}}^{2}$ is the hen's mate permanent environment variance, $\sigma_{pe_{Fno}}^{2}$ is the hen's permanent environment variance, $\sigma_{e_{ijkno}}^{2}$ is the error variance, A is the numerator relationship matrix, and I is an identity matrix.

### Cumulative Model

A cumulative model (CUM) that involves analyzing a single-point estimate of HoF using a cumulated average over the productive life of the hen was fitted with the following model:

[2] $$y_{ijk}=\mu +POU_{i}+Eg_{j}+a_{Fk}+e_{ijk}$$

The terms in model [2] are similar to those in model [1] with few modifications: $y_{ijk}$ is now the average of HoF over the productive life of the *k*^*th*^ animal belonging to the *i*^*th*^ POU fixed class with *j*^*th*^ average egg age, and $a_{Fk}$ is the random genetic effect of the animal (hen).

### Multitrait Model

The aim was to treat the repeated measurement as different traits. To avoid overparameterization due to the large numbers of measurements, the weekly records were divided into 3 parts on the basis of the pattern of the production curve. The pattern of hatchability tends to increase from the start of production up to a peak, which is sustained for a period of time after which it begins to decline. In essence, the HoF trait was categorized into 3 different traits classified as early, mid, and late HoF. The 3 different traits included average records from 27 to 37, 38 to 47, and 48 to 58 wk, for early, mid, and late traits, respectively. The MTM was as follows:

[3] $$y_{ijkm}=\mu +POU_{i}+Eg_{j}+a_{Fkm}+e_{ijkm}\text{,}$$

where $y_{ijkm}$ in this case is the observed record of the *k*^*th*^ animal for the *m*^*th*^ trait belonging to the *i*^*th*^ POU fixed class with *j*^*th*^ average egg age, and $a_{Fkm}$ is the random genetic effect of the *k*^*th*^ animal for the *m*^*th*^ trait; the other parameters are the same as mentioned in model [2].

### Fixed Regression Model

The REP as in model [1] was fitted but with a fixed productive curve fitted for HoF to account for the longitudinal nature of the trait using a fourth order Legendre polynomial. The FRM was as follows:

[4] $$y_{ijkno}=\mu +POU_{i}+Eg_{jk}+\overset{4}{\underset{j=1}{\sum }}b_{j}x_{jn}+a_{Fko}+pe_{Fko}+pe_{Mk}+e_{ijkno}$$

where $b_{j}$ is the fixed regression coefficient, and $x_{jn}$ is the incidence matrix value of the Legendre polynomials (Brotherstone et al., 2000) relating HoF to age. The other parameters are the same as those described in model [1].

### Random Regression Model

An extension of model [4] was carried out to model the deviation of each animal from the fixed production curve for HoF.

[5] $$y_{ijkno}=\mu +POU_{i}+Eg_{jk}+\overset{4}{\underset{j=1}{\sum }}b_{j}x_{jn}+\overset{2}{\underset{j=0}{\sum }}a_{Fko}x_{jn}+\overset{3}{\underset{j=0}{\sum }}pe_{Fko}x_{jn}+\overset{3}{\underset{j=0}{\sum }}pe_{Mk}x_{jn}+e_{ijkno}$$

where $x_{jn}$ is the incidence matrix value of the Legendre polynomial fitted for the additive genetic and permanent environment effects. The other terms were as described in model [1]. In this model, the temporary environment was modeled as heterogeneous with one class per week. This was done to adequately model the phenotypic variances at different ages. According to Olori et al. (1999), the residual error is highly variable at the early stages of production. With this, it was observed that heritability was biased upward in early production stages when comparing models with heterogeneous and constant residual variances using test day yield in dairy cattle. However, there was no observable effect on the estimates of genetic and permanent environment variances. The order of the Legendre polynomials used in this study was chosen because higher orders of the Legendre polynomials were computationally intense and did not converge.

### Threshold Model

Furthermore, HoF was additionally regarded as a binomial trait because the variances vary with the means. In this study, the standard deviations of HoF tended to decrease with increasing mean. Therefore, a threshold model might be more appropriate for this analysis, but this might not be easily fitted with an RRM. Therefore, a repeatability threshold model was fitted using a logit function in ASReml. The model was similar to model [1], but HoF was fitted as a proportion of the fertile eggs hatched while accounting for the total number of fertile eggs for each hen. Heritability in the liability scale were computed and transformed to an observable scale.

### Response to Selection

Genetic gain from improvement of a trait can be seen from the response to selection, and this is dependent on the accuracy of selection, additive genetic variances, and the intensity of selection, which is based on the proportion of selected individuals from a given population. The response to selection was estimated for each of the model used. A standardized selection differential of 50% for females was assumed in the calculation of responses mentioned in the following paragraphs. The following equations were used for the estimation of the expected response to selection based on a 50% standardized selection differential:

### For the CUM

[6] $$R=\frac{ir\sigma_{a}}{L}$$

where *R* is the response, r is the estimated selection accuracy, $\sigma_{a}$ is the additive genetic standard deviation, *i* is the intensity of selection, and *L* is the generation interval.

### For the RM, FRM, and RRM

[7] $$R=\frac{ir\sigma_{a}\sqrt{\frac{n}{1+\left(n-1\right)r_{e}}}}{L}$$

where *n* is the average number of observations per animal, $r_{e}$ is the repeatability estimated for the model [1,4,5], and the other parameters have been explained in the previous equation.

### For the MTM

A selection index was constructed and included all 3 traits with estimated genetic parameters.

[8] $$I=\overset{3}{\underset{i=1}{\sum }}b_{i}X_{i}$$

where *I* is the selection index, $X_{i}$ is the measurement for the *i*^*th*^ trait, and $b_{i}$ is the weight calculated from the inverse of the phenotypic (co)variance matrix P multiplied by the equivalent genetic (co)variance matrix G and a vector of economic weight a. In this study, equal economic weights were arbitrarily given to all 3 traits, and this could be changed by the breeding company depending on their breeding goals and what trait is deemed more economically important for higher emphasis.

[9] $$b=P^{-1}Ga$$

The variance of the selection index was estimated as $Var\left(I\right)=b^{\prime }Pb$, and the response per standardized selection differential is the square root of the variance of selection index.

[10] $$R=\frac{i\sqrt{var\left(I\right)}}{L}$$

A detailed explanation for response to selection for both single trait and multitrait can be found in the study by Cameron (1997).

## Results

**Figure 1:**
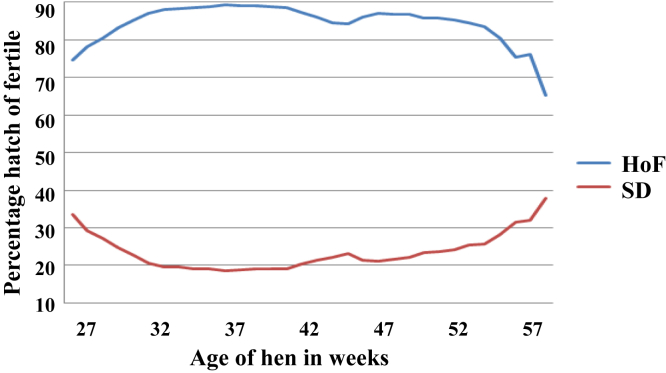
The trend of the percentage hatch of fertile (HoF) curve with blue curve indicating percent weekly average and the red curve indicating the standard deviation (SD) of percent HoF at various ages of the hen's productive life.

The mean and standard deviation of weekly percentage HoF trend are shown in Figure 1. This figure shows an increasing trend from the start of production at week 27 with an initial value of about 75%, which increased to approximately 90% at week 37 and remained constant until a gradual decline was observed at week 42. The drop in HoF reached a minimum of about 65% at the end of production, with a more steeply drop from week 57 to 58.

**Figure 2:**
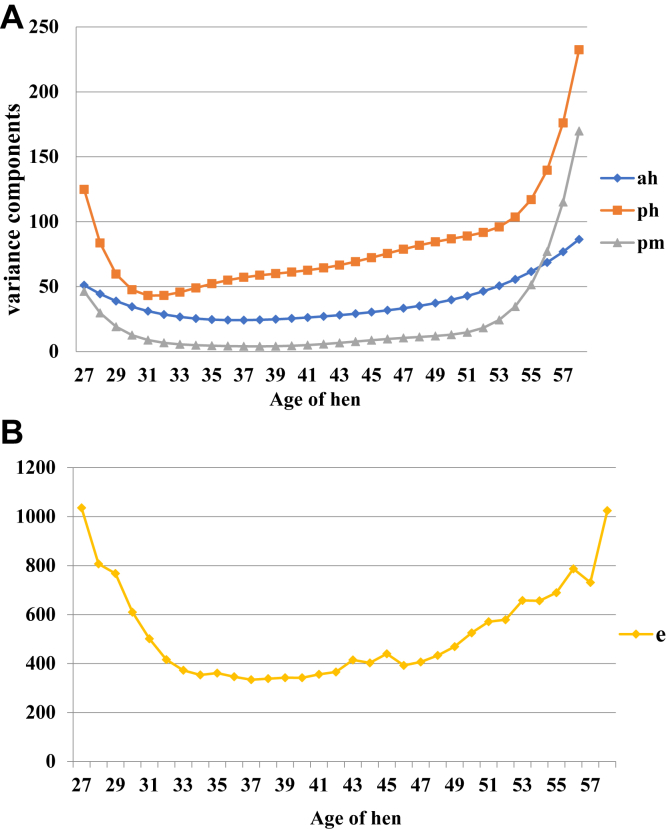
(A) Estimates of the hen genetic (ah) and permanent environment variances (ph) and the mate (sire) permanent environment variances (pm) from random regression model. (B) Residual variance estimates for each age of the hen using the random regression model.

The variance components estimated from the RRM are presented in Figures 2A and 2B. The figure indicates that the permanent environment variance of both the hen and her mate follow a similar trend, with an observable drop from the start of production after which a more constant level was found from week 31 to 52. Thereafter, an upward increase from week 53 to the end of the productive cycle of the animal was observed. On average, the permanent environment variance of the hen was 7 times more than that of her mate throughout the production cycle. For the additive genetic variance of the hen, a slight decline was found at early ages but then became almost constant for the production period with noticeable increase toward the end of production. The estimated residual variance from the RRM (Figure 2B) was observed to decrease at the early ages until 33 wk of age. Thereafter, it remained constant until at age 41 wk after which it increased with increasing age.

**Figure 3:**
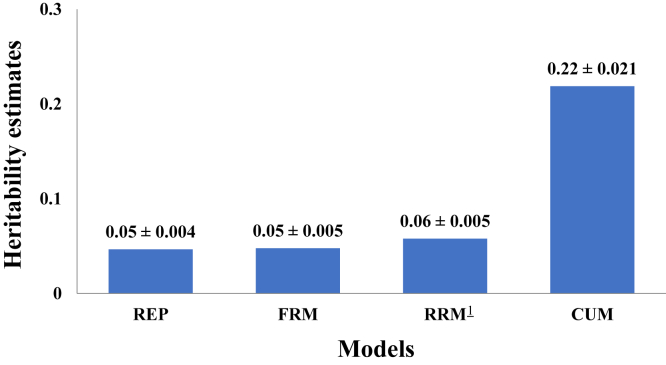
Heritability estimates and standard errors of hatch of fertile from the repeatability (REP), fixed regression (FRM), cumulative (CUM), and random regression (RRM) models. ^1^RRM estimate was the average across all ages.

**Figure 4:**
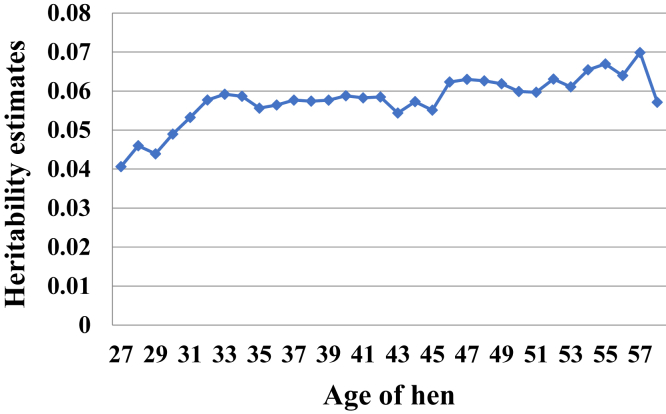
Heritability estimates for each age of the hen from a random regression model.

Heritability of HoF was estimated to be 0.22, 0.05, and 0.05 for univariate analysis using the CUM, REP, and FRM models, respectively (Figure 3). To facilitate comparison between all univariate analyses, average heritability for RRM was estimated at 0.06. For the simple REP, it was found that 8.31 and 1.05% of the phenotypic variance were accounted for by the permanent environment of the hen and her mate, respectively. The heritability of the 3 time points used as individual traits is presented in Table 2. Heritability for the early stage of production was found to be 0.14, while heritability estimates for mid and late stage of production were estimated to be 0.15 and 0.07, respectively. Figure 4 depicts the estimated heritability for each weekly age using RRM. These heritability estimates ranged from 0.04 to 0.07, with the lowest heritability being observed at the beginning of the productive cycle, whereas the highest heritability was observed at week 57. A liability heritability of 0.11 was obtained from the threshold model and was estimated to be 0.05 when transformed to an observable scale.

**Table 2:** Heritability ± standard error (diagonal), phenotypic correlation (lower triangle), and genetic correlation (upper triangle) estimates from the multitrait model.

Periods (weeks)	27–37	38–47	48–58
27–37	0.14 ± 0.02	0.86 ± 0.04	0.70 ± 0.09
38–47	0.36 ± 0.01	0.15 ± 0.02	0.94 ± 0.05
48–58	0.21 ± 0.01	0.36 ± 0.01	0.07 ± 0.01

Phenotypic and genetic correlations estimated from the MTM are presented in Table 2. Phenotypic correlations for the 3 traits analyzed ranged from 0.21 to 0.36. Conversely, the genetic correlation was higher than phenotypic correlation and ranged from 0.70 to 0.94. Generally, higher correlations were found between successive periods, while lower correlations were observed between periods farther apart. For RRM, the genetic correlations were estimated to range from 0.96 to 0.99 for adjacent ages (Table 3). However, as the ages got farther apart, these correlations decreased and varied from 0.36 to 0.93. The phenotypic correlations followed a similar pattern but were basically low for all ages and ranged from 0.01 to 0.25.

**Table 3:** Heritability^1^ (diagonal), phenotypic correlation (below diagonal), and genetic correlation (above diagonal) estimates from the random regression model.

Periods (weeks)	27	30	33	36	39	42	45	48	51	54	57
27	**0.041**	0.97	0.87	0.72	0.57	0.45	0.38	0.36	0.36	0.38	0.40
30	0.13	**0.049**	0.96	0.86	0.74	0.63	0.56	0.51	0.48	0.46	0.45
33	0.08	0.13	**0.059**	0.97	0.89	0.81	0.74	0.68	0.62	0.56	0.50
36	0.04	0.11	0.17	**0.056**	0.98	0.93	0.87	0.81	0.73	0.64	0.55
39	0.03	0.09	0.16	0.19	**0.057**	0.99	0.95	0.89	0.81	0.71	0.60
42	0.03	0.08	0.14	0.18	0.20	**0.058**	0.99	0.95	0.88	0.78	0.67
45	0.04	0.07	0.11	0.15	0.18	0.20	**0.055**	0.99	0.94	0.86	0.76
48	0.05	0.07	0.10	0.13	0.17	0.20	0.21	**0.062**	0.98	0.93	0.85
51	0.05	0.06	0.09	0.11	0.14	0.17	0.18	0.21	**0.060**	0.98	0.93
54	0.04	0.06	0.09	0.11	0.12	0.14	0.15	0.18	0.20	**0.065**	0.98
57	0.01	0.06	0.10	0.11	0.11	0.10	0.11	0.14	0.18	0.25	**0.070**

The estimated responses to selection based on the genetic parameters obtained from the models are presented in Table 4. Response to selection per year was estimated to be approximately 0.02, 0.03, 0.03, 0.07, and 0.07 for CUM, REP, FRM, MTM, and RRM, respectively. These genetic gains caused a percentage mean phenotypic value change that ranged from 0.03 to 0.08% in HoF. The highest mean value change in trait was found with the MTM, while the lowest resulted from the CUM.

**Table 4:** Genetic gain per year (ΔG) and their respective change in mean phenotypic values for hatch of fertile.^1^

Models	ΔG/year	Phenotypic mean^1^
Repeatability	0.025	3.03
Fixed regression	0.027	3.16
Cumulative	0.024	2.85
Multi trait	0.071	8.42
Random regression	0.071	8.41

1 Phenotypic mean was multiplied by 10^4^.

## Discussion

The mean hatchability of hens in this study was 84.40%, which is comparable to the 88.80% reported by Ledur et al. (2000).The slight difference may be due to the breed used in their study and the definition of hatchability, which in this study was defined as a proportion of fertile eggs that hatched. The trajectory pattern observed in the average weekly HoF is similar to those reported by Wolc et al. (2010). However, in this study, a more steep drop was found at the later ages, which could be attributed to the differences in the population or line as well as the age span of the hen used. In accordance to the study by Wolc et al. (2010), the estimated additive genetic effect of the hen's mate had no statistically significant contribution, and the heritability was less than 0.40%. This is understandable given that HoF in this study is the proportion of fertile eggs that hatched, and the impact of the male is limited once the egg is fertilized and the embryo start developing. Therefore, the hen's mate additive genetic effect was removed from subsequent models, and no heritability estimate was provided for the mate.

Fitness traits have been described to exhibit low heritability owing to their complex nature (Falconer and Mackay, 1996), and in the present study, there was no exception with heritability estimates ranging from 0.04 to 0.22 for the various models implemented. These estimates were in line with published literature values (Beaumont et al., 1997; Ledur et al., 2000; Sapp et al., 2004), with the exception of the CUM. The CUM has yet to be used for hatchability; therefore, the estimate of this model was compared with those estimated for egg production. In comparison with other models, the CUM always had the highest heritability estimate (Anang et al., 2000), which was also the case in this study. The high heritability obtained from the CUM could be as a result of the reduction in residual variance from averaging together highly variable records from all the different ages of the animal and assigning only a record to the animal. The simple averaging of the response variable in the CUM has not accounted for the correlated structure of the HoF recorded at various ages. This model also does not account for the permanent environment, which account for about 10% of the phenotypic variance for HoF. Wolc et al. (2007a) concluded that the CUM is not an appropriate model in adequately describing the egg production trait. Most traits observed and recorded in poultry are collected over the ages of the animal (longitudinal trend). These longitudinal patterns of the trait have been studied by models that take account of the changes that occur with the age of the animal (Laird and Ware, 1982). Hence, an REP and MTM are suitable for this purpose.

In the simple REP, heritability was assumed constant over the period of production, and the genetic correlations between ages were assumed equal to unity. This is contrary to the expression of different genes at different point in the ages of the animal, as well as the turning on and off of genes at different production periods (Swalve, 2000). The MTM and RRM allow for these differences in gene expression between different time periods to be accounted for in trait models. The heritability estimates were higher for the MTM than average heritability from the analogous RRM. Similar estimates for genetic parameters from monthly egg production in turkey were obtained when RRM was compared to MTM (Kranis et al., 2006). The higher heritability estimated from the MTM could have resulted from the inability of the model to account for the permanent environment effect of the animal, the time periods chosen as individual traits, and the uneven weighting of observations with age; that is, some animals had missing records for some time periods. Conversely, RRM estimated heritability for all the ages and predicted records for animals without records based on the information from the production trend of the animal. In addition, fitting heterogeneous residual variance reduced the overestimation of heritability that would have occurred at the start and end of production.

Fitness traits such as fertility and disease have been modeled using the threshold model (Bennewitz et al., 2007; Kapell et al., 2012b). This considers the trait as binary with 2 outcomes as either having the disease or not having the disease, as well as fertile or not fertile. This model estimates a liability heritability that could be transformed to the observed scale. The heritability estimated using the threshold model by Bennewitz et al. (2007) was higher than the estimates from the linear model. A higher liability heritability of 0.11 was obtained than the estimate from the REP. However, on the observed scale, the heritability of 0.05 was not very different from that of the linear REP. The higher heritability on the liability scale is in accordance with other studies (Kapell et al., 2012b). However, the computational time was 3 times more intensive than RRM. Thus, implementation on a routine basis in a production environment might not be feasible.

Correlations were estimated from both the RRM and MTM. The genetic correlations were higher than phenotypic correlations. This result is in accordance with the correlations estimated from test-day records in dairy cattle (Jamrozik and Schaeffer, 1997). In agreement with Jamrozik and Schaeffer (1997), closer ages had high correlations, and the correlations reduced with increasing distance between ages, indicating that closer ages could be treated as one trait and more distant ages as another trait.

The estimated response to selection shows the amount of genetic improvement expected to be achieved annually using the different models. The low response obtained from the CUM was not expected given that it had the highest heritability. However, the reason for this is that the model underestimates the phenotypic variance of the trait because of the inherent inadequacies of the model. In addition, the accuracy of selection was lower than that in other models, and the standard errors were higher than those in other models. Moreover, the lower standard errors estimated from the other models are due to the availability of more records per animal than a single record per animal when using the CUM. The MTM presented a higher response estimate than those estimated from the RRM; this might have resulted from the higher heritability from the MTM. The response estimated from the RRM could also have been confounded by the unequal number of the observations from the animal because this was a parameter used in estimating response for the RRM. The use of the (co)variance matrix used in MTM could also have resulted in the higher estimates.

## Conclusions

Although the CUM gave the highest heritability, it is not optimal for the modeling of genetic parameters for reproductive trait (HoF) measured over time. Owing to the fact that it causes the heritability estimates to be inflated and the response to selection to be deflated. The differences between the genetic correlations obtained from the MTM and RRM, particularly from more distant ages, suggest the appropriateness of these models over other models. While the study could have benefitted more from using the MTM in modeling the whole age trajectory of the animal, this would have been computationally infeasible because of the overparameterization of this model. Therefore, RRM was more robust than the other models in this study as estimates of parameters can be obtained for any age.

## Supplementary data

Author contributions

## References

[bib1] Abiola S.S., Meshioye O.O., Oyerinde B.O., Bamgbose M.A. (2008). Effect of egg size on hatchability of broiler chicks. Arch. Zootec..

[bib2] Anang A., Mielenz N., Schüler L. (2000). Genetic and phenotypic parameters for monthly egg production in White Leghorn hens. J. Anim. Breed. Genet..

[bib3] Anang A., Mielenz N., Schüler L. (2001). Monthly model for genetic evaluation of laying hens 1. Fixed regression. Br. Poult. Sci..

[bib4] Anang A., Mielenz N., Schüler L. (2002). Monthly model for genetic evaluation of laying hens II. Random regression. Br. Poult. Sci..

[bib5] Anang A., Mielenz N., Schüler L., Preisinger D.R. (2001). The use of monthly egg production records for genetic evaluation of laying hens. J. Ilmu Ternak Dan Vet..

[bib6] Avendaño S., Neeteson A.M., Fancher B. (2017). Broiler breeding for sustainability and welfare – are there trade-offs?, in. Proceedings of the New Zealand Poultry Beyond 2023 Conference.

[bib7] Beaumont C., Millet N., Le Bihan-Duval E., Kipi A., Dupuy V. (1997). Genetic parameters of survival to the different stages of embryonic death in laying hens. Poult. Sci..

[bib8] Bennewitz J., Morgades O., Preisinger R., Thaller G., Kalm E. (2007). Variance component and breeding value estimation for reproductive traits in laying hens using a Bayesian threshold model. Poult. Sci..

[bib9] Brito L.F., Silva F.G., Oliveira H.R., Souza N.O., Caetano G.C., Costa E.V., Menezes G.R.O., Melo A.L.P., Rodrigues M.T., Torres R.A. (2017). Modelling lactation curves of dairy goats by fitting random regression models using Legendre polynomials or B-splines. Can. J. Anim. Sci..

[bib10] Brotherstone S., White I.M.S., Meyer K. (2000). Genetic modelling of daily milk yield using orthogonal polynomials and parametric curves. Anim. Sci..

[bib11] Cameron N.D. (1997). Selection Indices and Prediction of Genetic Merit in Animal Breeding.

[bib12] Chambers J.R., Crawford R.D. (1990). Genetics of growth and meat production in chickens. Poultry Breeding and Genetics.

[bib13] Decuypere E., Bruggeman V., Barbato G.F., Buyse J. (2003). Growth and reproduction problems associated with selection for increased broiler meat production. Poultry Genetics, Breeding and Biotechnology.

[bib14] Executive Guide to World Poultry Trends, A (2019). Poultry Trends - Poultry Trends 2019.

[bib15] Falconer D.S., Mackay T.F.C. (1996). Introduction to Quantitative Genetics.

[bib16] FAO (2006). Interim Report Prospects for Food, Nutrition, Agriculture and Major Commodity Groups. Food and Agriculture Organization of the United Nations.

[bib17] Gilmour A.R., Gogel B.J., Cullis B.R., Thompson R. (2009). ASReml User Guide Release 3.0.

[bib18] Heier B.T., Jarp J. (2001). An epidemiological study of the hatchability in broiler breeder flocks. Poult. Sci..

[bib19] Jamrozik J., Schaeffer L.R. (1997). Estimates of genetic parameters for a test day model with random regressions for yield traits of first lactation Holsteins. J. Dairy Sci..

[bib20] Kapell D.N.R.G., Hill W.G., Neeteson A.-M., McAdam J., Koerhuis A.N.M., Avendaño S. (2012). Twenty-five years of selection for improved leg health in purebred broiler lines and underlying genetic parameters. Poult. Sci..

[bib21] Kapell D.N.R.G., Hill W.G., Neeteson A.-M., McAdam J., Koerhuis A.N.M., Avendaño S. (2012). Genetic parameters of foot-pad dermatitis and body weight in purebred broiler lines in 2 contrasting environments. Poult. Sci..

[bib22] Kheirabadi K. (2018). Bayesian analysis of random regression models to model test-day somatic cell score of primiparous holstein cattle in Iran. J. Appl. Anim. Res..

[bib23] Koerhuis A.N.M., McKay J.C. (1996). Restricted maximum likelihood estimation of genetic parameters for egg production traits in relation to juvenile body weight in broiler chickens. Livest. Prod. Sci..

[bib24] König S., Köhn F., Kuwan K., Simianer H., Gauly M. (2006). Use of repeated measures analysis for evaluation of genetic background of dairy cattle behavior in automatic milking systems. J. Dairy Sci..

[bib25] Kranis A., Hocking P.M., Hill W.G., Woolliams J.A. (2006). Genetic parameters for a heavy female Turkey line: impact of simultaneous selection for body weight and total egg number. Br. Poult. Sci..

[bib26] Laird N.M., Ware J.H. (1982). Random-effects models for longitudinal data. Biometrics.

[bib27] Lapão C., Gama L.T., Soares M.C. (1999). Effects of broiler breeder age and length of egg storage on albumen characteristics and hatchability. Poult. Sci..

[bib28] Laughlin K. (2007). The evolution of genetics, breeding and production. Tempert. Fellowsh. Rep..

[bib29] Ledur M.C., Fairfull R.W., Mcmillan I., Gowe R.S., Asselstine L. (2000). Genetic effects of ageing on fertility and hatchability in the first laying cycle of three White Leghorn strains and their two-way crosses. Br. Poult. Sci..

[bib30] Liljedahl L.-E., Fairfull R.W., Gowe R.S. (1999). Age-regulated expression of genetic and environmental variation in fitness traits. 1. Genetic effects and variances for egg production in a factorial mating of six selected Leghorn strains. Can. J. Anim. Sci..

[bib31] Luo P.T., Yang R.Q., Yang N. (2007). Estimation of genetic parameters for cumulative egg numbers in a broiler dam line by using a random regression model. Poult. Sci..

[bib32] Miyumo S., Wasike C.B., Kahi A.K. (2018). Genetic and phenotypic parameters for feed efficiency in indigenous chicken in Kenya. Livest. Sci..

[bib33] OECD/FAO (2018). *OECD - FAO Agricultural Outlook 2018 – 2027.* Paris: OECD Publishing. 10.1787/agr_outlook-2018-en.

[bib34] Olori V.E., Hill W.G., Brotherstone S. (1999). The structure of the residual error variance of test day milk yield in random regression models. Interbull Bull.

[bib35] Padilha A.H., Alfonzo E.P.M., Daltro D.S., Torres H.A.L., Neto J.B., Cobuci J.A. (2019). Genetic trends and genetic correlations between 305-day milk yield, persistency and somatic cell score of Holstein cows in Brazil using random regression model. Anim. Prod. Sci..

[bib36] Permin A., Pedersen G. (2000). Problems related to poultry production at village level. Possibilities for Smallholder Poultry Projects in Eastern and Southern Africa.

[bib37] Ptak E., Schaeffer L.R. (1993). Use of test day yields for genetic evaluation of dairy sires and cows. Livest. Prod. Sci..

[bib38] R Core Team, R: A Language and Environment for Statistical Computing (RDC Team, Ed.) 1. 2012. R Foundation for Statistical Computing, Vienna, Austria. Accessed Mar. 2021. www.R-project.org.

[bib39] Santana M.L., Eler J.P., Bignardi A.B., Ferraz J.B.S. (2015). Two-trait random regression model to estimate the genetic association of scrotal circumference with female reproductive performance in Nelore cattle. Theriogenology.

[bib40] Sapp R.L., Rekaya R., Misztal I., Wing T. (2004). Male and female fertility and hatchability in chickens: a longitudinal mixed model approach. Poult. Sci..

[bib41] Schaeffer L.R. (2004). Application of random regression models in animal breeding. Livest. Prod. Sci..

[bib42] Schaeffer L.R., Dekkers J.C.M. (1994). Random regressions in animal models for test-day production in dairy cattle. Proceedings of the 5th World Congress on Genetics Applied to Livestock Production.

[bib43] Swalve H.H. (1995). The effect of test day models on the estimation of genetic parameters and breeding values for dairy yield traits. J. Dairy Sci..

[bib44] Swalve H.H. (2000). Theoretical basis and computational methods for different test-day genetic evaluation methods. J. Dairy Sci..

[bib45] VanVleck L.D., Doolittle D.P. (1964). Genetic parameters of monthly egg production in the Cornell Controls. Poult. Sci..

[bib46] Wilson H.R. (1997). Effects of maternal nutrition on hatchability. Poult. Sci..

[bib47] Wolc A., Lisowski M., Szwaczkowski T. (2007). Genetic evaluation of laying hens under fixed regression animal models. Arch. Anim. Breed..

[bib48] Wolc A., Lisowski M., Szwaczkowski T. (2007). Heritability of egg production in laying hens under cumulative, multitrait and repeated measurement animal models. Czech J. Anim. Sci..

[bib49] Wolc A., Szwaczkowski T. (2009). Estimation of genetic parameters for monthly egg production in laying hens based on random regression models. J. Appl. Genet..

[bib50] Wolc A., White I.M.S., Hill W.G., Olori V.E. (2010). Inheritance of hatchability in broiler chickens and its relationship to egg quality traits. Poult. Sci..

[bib51] Wolc A., White I.M., Olori V.E., Hill W.G. (2009). Inheritance of fertility in broiler chickens. Genet. Sel. Evol..

