# Supplementary material for: Modeling genetic components of hatch of fertile in broiler breeders
Source: Poult Sci. 2021 Feb 19;100(5):101062. doi: 10.1016/j.psj.2021.101062 (PMC8008174; doi:10.1016/j.psj.2021.101062)
Supplement: Author contributions [file mmc1.docx]

Authors' contributions:

B.O.M. carried out the analyses, interpreted the results, and wrote the manuscript. V.E.O. conceived the idea of the project. V.E.O. and R.A.M. supervised the analyses, participated in the design of the study, and revised the manuscript. All authors read and approved the final manuscript.
